# A perfect match between borophene and aluminium in the AlB_3_ heterostructure with covalent Al–B bonds, multiple Dirac points and a high Fermi velocity†

**DOI:** 10.1039/d1sc05207a

**Published:** 2021-12-20

**Authors:** Yalong Jiao, Fengxian Ma, Xiaolei Zhang, Thomas Heine

**Affiliations:** Faculty for Chemistry and Food Chemistry, TU Dresden Bergstraße 66c 01069 Dresden Germany thomas.heine@tu-dresden.de; College of Physics, Hebei Key Laboratory of Photophysics Research and Application, Hebei Normal University Shijiazhuang 050024 China; Helmholtz-Center Dresden Rossendorf, Institute of Resource Ecology, Leipzig Research Branch 04316 Leipzig Germany; Department of Chemistry, Yonsei University 03722 Seoul Korea

## Abstract

By performing a swarm-intelligent global structure search combined with first-principles calculations, a stable two-dimensional (2D) AlB_3_ heterostructure with directed, covalent Al–B bonds forms due to a nearly perfect lattice match between 2D borophene and the Al(111) surface. The AlB_3_ heterosheet with the *P*6*mm* space group is composed of a planar Al(111) layer and a corrugated borophene layer, where the in-plane coordinates of Al covalently link with the corrugated B atoms. The resulting structure shows a similar interlayer interaction energy to that of the Al(111) surface layer to the bulk and high mechanical and thermal stability, possesses multiple Dirac points in the Brillouin zone with a remarkably high Fermi velocity of 1.09 × 10^6^ m s^−1^, which is comparable to that of graphene. Detailed analysis of the electronic structure employing the electron localisation function and topological analysis of the electron density confirm the covalent Al–B bond with high electron localisation between the Al and B centres and with only little interatomic charge transfer. The combination of borophene with metal monolayers in 2D heterostructures opens the door to a rich chemistry with potentially unprecedented properties.

## Abstract 2

By performing a swarm-intelligent global structure search combined with first-principles calculations, a stable 2D AlB_3_ heterostructure with directed, covalent Al–B bonds forms due to a nearly perfect lattice match between 2D borophene and the Al(111) surface.

## Graphical abstract

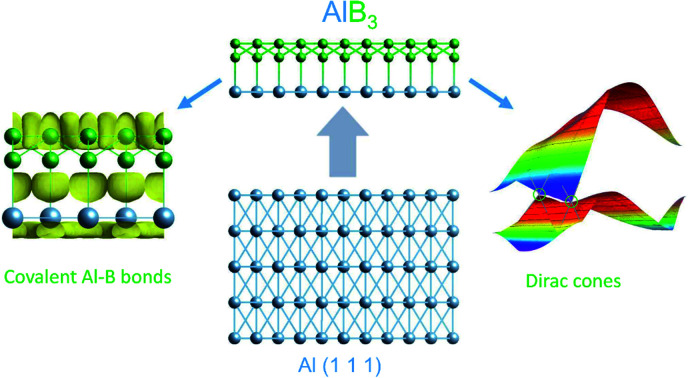

## Introduction

Two-dimensional (2D) heterostructures offer a plethora of opportunities to develop diverse functional devices by integrating highly disparate atomically-thin layers without considering the atomic commensurability such as that in layered bulk allotropes.^1^ Their unique structures provide a full range of intriguing properties and make the heterostructures widely used as an essential building block for the next generation of nanoscale devices.^2^

Borophene,^3,4^ the 2D form of boron, has received great attention due to its intriguing characteristics such as excellent transport properties,^5,6^ superconductivity,^7,8^ mechanical compliance,^9^ and optical transparency.^10^ As a trivalent element, boron exhibits structural polymorphism, especially in 2D limits,^11^ thus providing versatile platforms to stack heterostructures owing to their diverse bonding configurations.^12,13^ However, studies of integrating heterostructures with borophene sheets are primarily limited. This is partially because the fabrication of borophene significantly relies on metal surfaces and these substrates display an active catalytic reaction with the overlayer, making it rather challenging to transfer a freestanding borophene sheet to form a heterostructure. Indeed, an active reaction at the metal surface sometimes can induce the realisation of novel 2D crystals. A typical example is PtSe_2_,^14^ in which direct selenization of the Pt (111) surface will trigger the reaction to form a noble metal dichalcogenide.^15^ Recently, metal surfaces were also utilised to grow pure 2D metals.^16^ Considering the crucial role of metal substrates in fabricating 2D materials, it is of high interest to explore whether novel heterostructures can be realised when borophene is grown on various metal surfaces.

The covalent bond typically provides the strongest directed link between two or more atoms by sharing valence electrons, resulting in a new electron orbit. Although covalent bonds are usually formed by non-metal elements, exceptions can be found when metal elements are combined with highly electronegative elements such as S or F, as manifested for example in the CuS crystal or WF_6_ molecule.^17,18^ When metals are combined with electropositive elements (such as group 13 elements) which tend to lose electrons during bonding, the formation of covalent bonds becomes rather challenging.

In this work, we demonstrate that the deposition of an all-boron layer on a clean Al(111) surface results in the formation of a stable AlB_3_ heterosheet with covalent bonds between Al and B. According to our density-functional theory (DFT) calculations, the estimated cleavage energy to break the Al–B bonds is 1.80 J m^−2^, comparable to the interaction energy between two Al(111) layers (1.89 J m^−2^), which suggests that the material can be made by deposition of boron on a clean Al(111) surface followed by mechanical exfoliation (Fig. S1†).^19^ Band structure calculations show that the AlB_3_ heterosheet is semimetallic with multiple Dirac cones in the Brillouin zone, indicating intriguing electronic properties. This work proposes an effective strategy towards a metal-borophene heterostructure and suggests a new way of forming heterostructures by atomic deposition on metal surfaces.

## Methods

The structural search was performed based on the particle swarm optimization (PSO) technique as implemented in the crystal structure analysis by using the particle swarm optimization (CALYPSO) code.^20–22^ The population size was set to 30, and 60% of structures in each generation were evolved into the next generation by PSO. The structural relaxations were performed on the basis of the density functional theory (DFT) as implemented in the Vienna *ab initio* Simulation Package (VASP).^23,24^ The electronic exchange–correlation functional was treated by the generalized gradient approximation (GGA) proposed by Perdew, Burke and Ernzerhof (PBE).^25^ The energy cutoff of the plane wave basis was set to 400 eV. The structures were fully relaxed until the maximum force on each atom was less than 5 meV Å^−1^. The energy convergence criterion in the self-consistent calculations was set to 10^−6^ eV. A Gamma-centered Monkhorst–Pack *k*-point mesh of 13 × 13 × 1 was used for geometry optimization and self-consistent calculations. A vacuum slab of ∼15 Å in the *z* direction was adopted to avoid artificial interactions between the neighboring layers. The dipole correction has a negligible influence on the energy and structure of the AlB_3_ heterosheet and was turned off in the production calculations. The band dispersions were confirmed by using the GW (G_0_W_0_) approximation. The phonon dispersion was computed by using the Phonopy code^26^ within the density functional perturbation theory (DFPT).^27^ To evaluate the thermal stability of the structure, *ab initio* molecular dynamics (AIMD) simulations with a 6 × 6 supercell were performed. The visualization of the structure and of the electron localization function (ELF) were carried out with VESTA.^28^ Bader analysis was performed within the DFT/PBE/TZP basis set using the BAND code.^29,30^ The bond order analysis was performed by using the density derived electrostatic and chemical (DDEC) method.^31^

## Results and discussion

### Structure

**Fig. 1:**
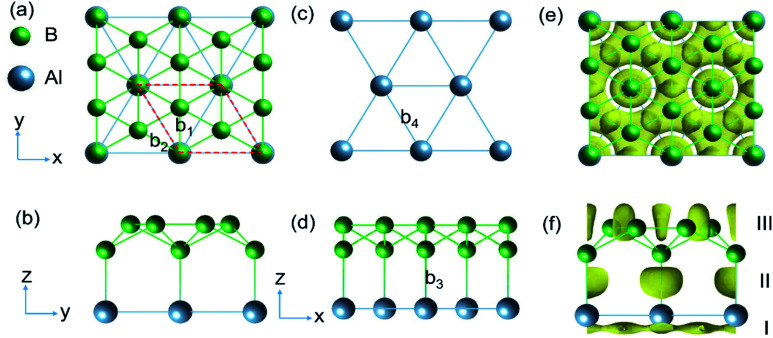
(a, b and d) Top and side view of the AlB_3_ heterosheet. The red dashed lines in (a) represent the primitive cell of the layer. (c) Al (111) sublayer. (e and f) Corresponding electron localization function (ELF) with an isovalue of 0.75. Three ELF domains are marked by Roman numerals in (f) and discussed in the text.

The structure of AlB_3_ (Fig. 1) is composed of a planar Al layer with the geometry as for the Al(111) surface layer and a corrugated borophene-like sublayer,^32^ with a corrugation pattern resembling that of the well-known structure of B_7_ clusters (in anionic, cationic and neutral forms)^33,34^ (Fig. 1c). The AlB_3_ heterosheet crystallizes in the *P*6*mm* space group with a lattice constant of *a* = *b* = 2.845 Å. The primitive cell contains 1 Al and 3 B atoms and the B–B bond lengths are *b*_1_ = 1.87 Å and *b*_2_ = 1.64 Å (Fig. 1a), which are comparable to that in the B_7_ cluster (1.56–1.76 Å)^35^ and *Pmmn* phase of borophene (1.62–1.87 Å).^36^ The Al–Al bond length is 2.845 Å, very close to that in the Al solid (2.856 Å), resulting in a lattice mismatch of 0.2% between the Al sublayer in the AlB_3_ heterosheet and the Al (111) surface. The B–Al bond length in AlB_3_ is 2.17 Å, which is smaller than that in the three-dimensional *P*6/*mmm* phase AlB_2_ crystal (2.39 Å, Fig. S2a†).^37^ This short bond length already suggests a high Al–B bond strength in the AlB_3_ heterosheet. To further test whether the Al–B bond is robust against external forces, the interlayer binding energy *E*_il_ was calculated. The *E*_il_ is defined as:*E*_il_ = (*E*_tot_ − *E*_Al_ − *E*_B_)/*S*where *E*_tot_,*E*_Al_ and *E*_B_ represent the total energy of the fully relaxed unit cell, Al sublayer and B sublayer, respectively. S is the area of the sheet. The absolute value of E_il_ is estimated to be 1.80 J m^−2^, which is one order of magnitude larger than that in graphite (0.19 J m^−2^)^38^ and indicated the strong interaction between Al and B sublayers. In comparison, the Al(111) surface layer shows a cleavage energy of 1.89 J m^−2^. The comparable *E*_il_ between AlB_3_ and the Al(111) surface indicates that exfoliation of a single AlB_3_ sheet from the Al metal surface is possible.

### Bonding analysis

First, we analyse the bonding behaviour in AlB_3_ by employing the real space electron localization function (ELF).^39,40^ The ELF ranges from 0 to 1, where 0 means a low electron density, 1 indicates the perfect localization of electrons and 0.5 implies the presence of a free electron gas.^38^ To test the reliability of this method, we calculated the ELF for the previously reported covalent Cu–S bond in the CuS crystal.^18^ As shown in Fig. S2,† the electron localisation between Cu and S atoms occurs to a large extend (ELF attains a maximum value of 0.90) and confirms the covalent bonding nature and the reliability of this approach.

As shown in Fig. 1e and f, the ELF of the AlB_3_ heterosheet exhibits three main domains: Domain I presents a 2D electride-like density distribution below the Al sublayer, suggesting metallic bonding within the Al layer. Domain II in between the Al–B layers is characterized by localized electron pockets which indicate the covalent bonding states (σ-bonds). Domain III localises within the boron sublayer, implying the covalent B–B bonding states. For comparison, we tested the Al–B bonding character in the AlB_2_ crystal by plotting the corresponding ELF as presented in Fig. S3a,† which shows the absence of electron localisation between Al and B centers in this predominant ionic solid.

**Fig. 2:**
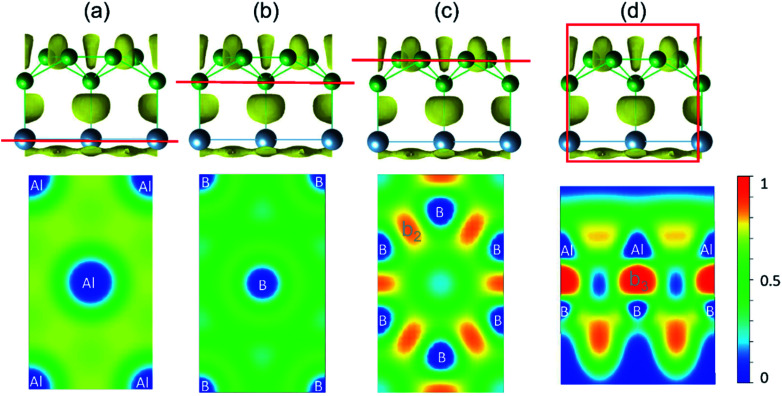
(a–d) Side view and slab cut along the (001) direction of the ELF with an isovalue of 0.75 (top panel). Red lines indicate selected layers in ELF contour maps shown in the bottom panel.

The bonding character within AlB_3_ becomes evident if ELF-slices through potential bonds are analysed (Fig. 2a–d). However for the hexagonal Al (Fig. 2a) and B (Fig. 2b) layers the diffuse ELF suggests only weak interactions, and a remarkably high electron concentration is present in the honeycomb B layer (Fig. 2c) and between the Al–B atoms (Fig. 2d), confirming the strong covalent bonding nature between the adjacent Al and B centers. The unique structural configuration of AlB_3_ can be regarded as a prototype to further explore other metal–boron systems. In contrast, the ELF for isoelectronic GaB_3_ and InB_3_ heterosheets (Fig. S3†) indicates polar covalent bonds between the metal and boron atoms.

**Fig. 3:**
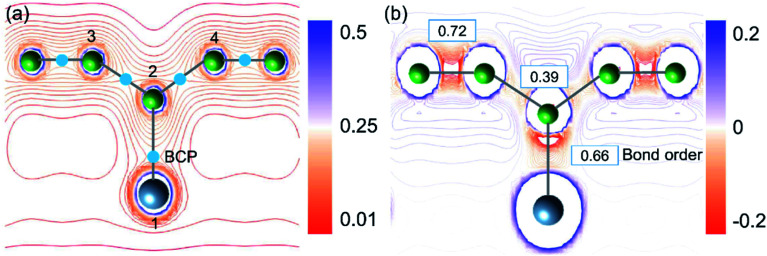
(a) Cut plane of the valence electron density for the AlB_3_ heterosheet. Light-blue dots: bond critical points (BCP). (b) Cut plane of the Laplacian of the electron density. The positive values indicate local charge depletion (blue) and negative values indicate local charge accumulation (red). Blue frames: the values of the bond order.

To validate the conclusions of the ELF analysis, we applied the topological analysis of the electron density based on the quantum theory of atoms in molecules (QTAIM) to the AlB_3_ heterosheet. The bond critical points (BCPs), which correspond to saddle points in the electron density distribution, were calculated and are depicted in Fig. 3a. They confirm the covalent framework between boron next neighbours and in particular between Al and the corrugated B atoms. The BCP appears between adjacent aluminium atoms as well, but is not shown in the current cut plane.

As charge analyses tend to be somewhat ambiguous, we employed three substantially different methods to analyse the charge redistribution in AlB_3_ (Table 1), *i.e.* Hirshfeld charges, Voronoi deformation charges, and Charge Model 5 (CM5). In agreement, all three methods manifest that the electron transfer between Al (1) and B (2) atoms is negligible, and the small loss of electron from Al (1) is mainly received by B (3) and B (4) atoms. A quantitative atomic population analysis reveals that the Al–B (b_3_) bond order is 0.66 which is comparable to that of the Al–B (b_2_) bond (0.72) in the honeycomb boron layer, indicating their analogous covalent bonding behaviour. The bond order of Al–B is even larger than that in the diborane (B_2_H_6_) molecule whose 3c–2e bond exhibits a value of 0.5. In contrast, the bond order of B–B (b_1_ in Fig. 1a) and Al–Al atoms (b_4_, in Fig. 1c) are relatively low (0.39 and 0.36, respectively), reflecting their delocalised electron states.

**Table 1:** The charge analysis of the AlB_3_ heterolayer by different methods. Atom numbers correspond to the labelling shown in Fig. 3a

Methods	Atoms			
	Al(1)	B(2)	B(3)	B(4)
Hirshfeld charges	0.062	0.002	−0.032	−0.032
Voronoi deformation charges	0.057	0.038	−0.048	−0.048
Charge model 5	0.094	−0.025	−0.034	−0.034

The Laplacian (∇^2^*ρ*(*r*), the spatial second derivative) of the electron density (*ρ*(*r*)) provides a measure of the local electron concentration or depletion, which is a key indicator of bond formation. Fig. 3a demonstrates that the electrons are predominantly accumulated along the Al–B and B–B bond paths. In general, the small charge transfer between Al and B atoms together with the electron accumulation along the Al–B bond path is a solid evidence for the covalent Al–B bonds.

### Chemical activity towards molecular adsorption

With an electride-like density distribution in the vicinity of the Al sublayer, it is interesting to evaluate the chemical activity of the AlB_3_ heterosheet through the adsorption of H_2_, H_2_O and O_2_ molecules. In our calculations, the molecules were placed on the Al surface with a small distance (∼1.8 Å) before geometry optimization. The fully relaxed configurations are shown in Fig. S4,† where we can find that the layer is inert to H_2_ and H_2_O adsorption. The distance between H_2_ (H_2_O) and the heterosheet is 3.50 (2.24) Å, indicating the physisorption of the two molecules. In comparison, the O_2_ molecule undergoes dissociative adsorption on the AlB_3_ heterosheet and forms chemical bonds with the Al surface, indicating a similar surface oxidation that is known from bulk Al surfaces. However, by plotting the corresponding ELF of the AlB_3_ + O_2_ and fully oxidized AlB_3_ systems (Fig. S4 and S5†), we can clearly see that oxidation does not affect the covalent bonding nature of the Al–B bonds.

### Electronic properties

**Fig. 4:**
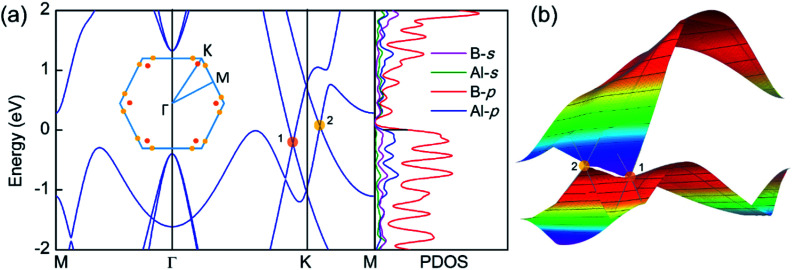
(a) The band structure and projected density of states (PDOS) of the AlB_3_ heterosheet. Insets: The Brillouin zone where the positions of different Dirac points are shown by orange and yellow dots. (b) 3D band dispersion of the Dirac point 1 and 2.

The electronic band structure and the corresponding projected density of states (PDOS) were calculated to evaluate the electronic performance of the AlB_3_ heterosheet and are shown in Fig. 4. Valence and conduction bands meet near the Fermi level and form two cone shaped Dirac points along the *Γ*–*K* and *K*–*M* lines. DP1 along the *Γ*–*K* line is 0.2 eV below the Fermi level, while DP2 along the *K*–*M* line is 0.07 eV above the Fermi level. The Dirac points are also preserved at a more advanced GW level (Fig. S6†). The calculated PDOS is close to zero at the Fermi level. Moreover, a direct band gap of 1.7 eV is found at the *Γ* point. The Fermi velocities 
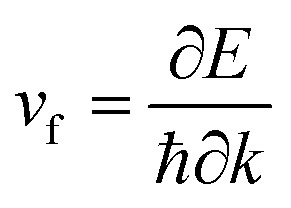
 were calculated to be 1.09 × 10^6^ m s^−1^ for DP1 and 6.4 × 10^5^ m s^−1^ for DP2. These values are comparable to that in graphene (∼10^6^ m s^−1^). By plotting the 3D band dispersions, we found that the Dirac points are actually conical shaped in the reciprocal space, which is analogous to that in graphene. By considering the effect of the symmetry, there would be six cones like DP1 and twelve cones like DP2 (Fig. S7†) in the first Brillouin zone. Notably, the Dirac points are robust when tensile strain is applied. (Fig. S8†) The multiple Dirac cones enable the excellent electron transport properties of the heterosheet.

**Fig. 5:**
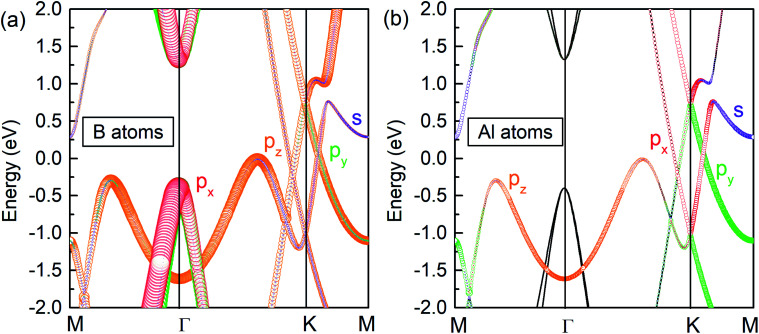
Orbital resolved band structure of (a) B and (b) Al atoms in the AlB_3_ heterosheet.

To explore the origin of the Dirac states, the orbital-resolved band structures were calculated. As shown in Fig. 5, the dominant contribution to the valence band maximum and conduction band minimum at the *Γ*-point originates from the p_*x*_ and p_*y*_ orbitals of B atoms (Fig. 5a). The formation of DP1 is mainly attributed to the hybridization of the B-p_*z*_ orbital and Al-p_*x*_ and Al-p_*y*_ orbitals while that of DP2 is attributed to the hybridization of B-p_*y*_, B-p_*z*_, Al-p_*x*_ and Al-p_*y*_ states. The detailed orbital weights at the Dirac points are summarised in Table 2.

**Table 2:** Weights of different orbitals in the AlB_3_ heterosheet at Dirac point (DP) 1 and 2

Elements	Orbitals			
	S	P_*x*_	P_*y*_	P_*z*_
B (DP 1)	0.035	0.039	0.014	0.184
B (DP 2)	0.024	0.004	0.055	0.194
Al (DP 1)	—	0.071	0.019	—
Al (DP 2)	—	0.085	0.11	—

### Stability

A mechanically stable 2D hexagonal lattice should satisfy the following elastic stability criteria:^41^ C_11_ >0, C_11_ × C_22_ >C_12_^2^, and C_66_ >0, where C_ij_ are the elastic constants. The values summarised in Table S1† fully meet the criteria, confirming the mechanical stability of the AlB_3_ heterostructure. Besides, no imaginary frequencies are found in the phonon dispersion (Fig. S9†), indicating its kinetic stability. The highest phonon frequencies of the layer reach up to 26.7 THz (890.6 cm^−1^), which is higher than the highest frequencies found in silicene (580 cm^−1^),^42^ Cu_2_Si (420 cm^−1^)^43^ and MoS_2_ monolayers (473 cm^−1^).^44^ Such high-energy phonons characterize the robust bond interactions in the layer. Interestingly for surface science studies, the borophene layer maintains this stable configuration even if stacked on the Al(111) bilayer or trilayer (Fig. S10†). AIMD simulation was performed to confirm the thermal stability of the heterostructure. The snapshots after 10 ps of simulated annealing at 300 K are presented in Fig. S11.† We can clearly find the structure to be well maintained, indicating that it is thermally stable. It should be noted that our structure search has also generated several AlB_3_ allotropes (Fig. S12 and S13†). After carefully examining their energies and stabilities, we confirm that the AlB_3_ heterosheet is the most stable configuration.

## Conclusions

A novel AlB_3_ heterosheet can be formed when a borophene layer deposited on the Al(111) surface as reported in ref. 11 is exfoliated. Exfoliation of AlB_3_ from the Al surface is possible due to the strong covalent Al–B bonds. The isolated AlB_3_ sheet shows high thermal, dynamic and mechanical stabilities. As can be seen from the band structure calculations, it displays multiple Dirac points with an ultra-high Fermi velocity comparable to that in graphene. We elaborated that borophene combined with a pure metal layer is able to form intriguing heterosheets with unusual chemical bonding states and enhanced electronic performance. This work enriches the diversity of 2D heterostructures with exotic properties.

## Data availability

Structural data is available at the NOMAD repository with material ID lymrPBgwnvfSacCF2fLnIF8rRkAA. The direct link is https://nomad-lab.eu/prod/rae/gui/entry/id/ijb2DdMWQziD9_8AQxNh-Q/WwHmihRyxyiOpwBMVpzqROIBmto6.

## Author contributions

TH and YJ conceived the project. YJ, FM and XZ performed calculations. TH, YJ and FM analyzed the data and wrote the paper. All authors commented on the manuscript.

## Conflicts of interest

There are no conflicts to declare.

## Supplementary Material

SC-013-D1SC05207A-s001
