# Supplementary material for: A perfect match between borophene and aluminium in the AlB3 heterostructure with covalent Al–B bonds, multiple Dirac points and a high Fermi velocity
Source: Chem Sci. 2021 Dec 20;13(4):1016–22. doi: 10.1039/d1sc05207a (PMC8790795; doi:10.1039/d1sc05207a)
Supplement: SC-013-D1SC05207A-s001 [file SC-013-D1SC05207A-s001.pdf]

Electronic Supplementary Material (ESI) for Chemical Science.
This journal is © The Royal Society of Chemistry 2021

Electronic Supplementary Information

Perfect match between borophene and aluminium in AlB_3 heterostructure with covalent Al-B bonds, multiple Dirac points and high Fermi velocity

Yalong Jiao[1,#], Fengxian Ma[2,#], Xiaolei Zhang[2], Thomas Heine[1,3,4,*]

[1]Faculty for Chemistry and Food Chemistry, TU Dresden, Bergstraße 66c, 01069 Dresden, Germany

[2]College of Physics, Hebei Key Laboratory of Photophysics Research and Application, Hebei Normal University, 050024 Shijiazhuang, China

[3]Helmholtz-Center Dresden Rossendorf, Institute of Resource Ecology, Leipzig Research Branch, 04316 Leipzig, Germany

[4]Department of Chemistry, Yonsei University, 03722 Seoul, Korea

*E-mail: thomas.heine@tu-dresden.de

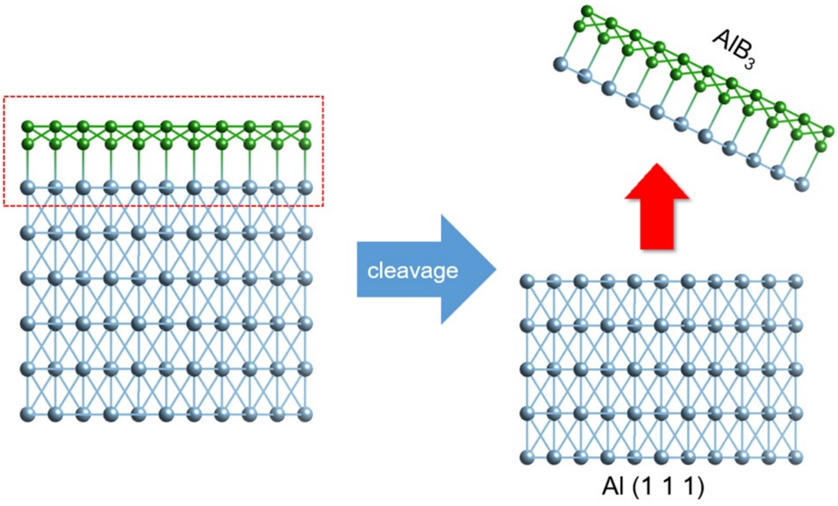

Fig. S1 Illustration of cleaving the AlB_3 structure from the Al (1 1 1) surface.

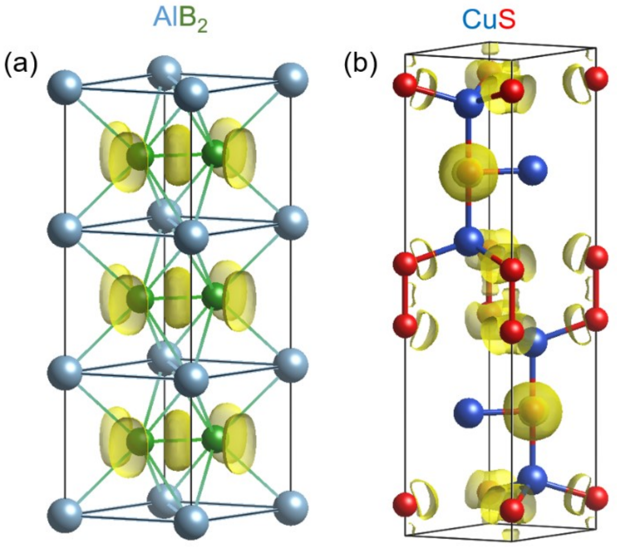

Fig. S2 ELF of the experimentally realised (a) AlB_2 and (b) CuS crystal. The isosurface is set to 0.75.

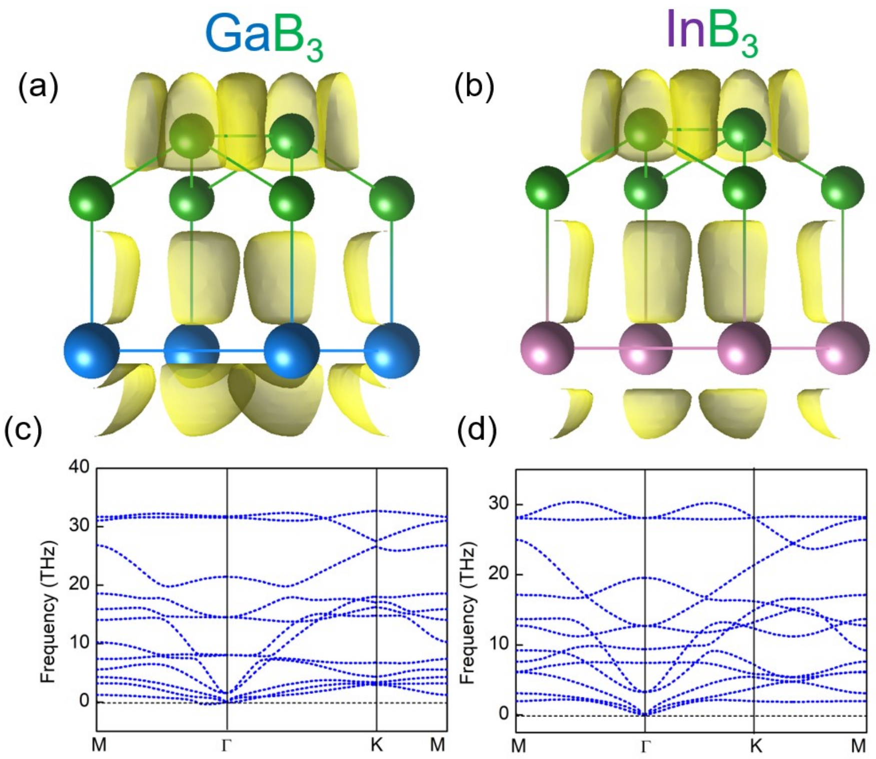

Fig. S3 (a-b) ELF of the GaB_3 and InB_3 heterostructures, where the covalent bands can be seen between the metal and boron atoms. (c-d) The phonon spectrum for the two heterolayers.

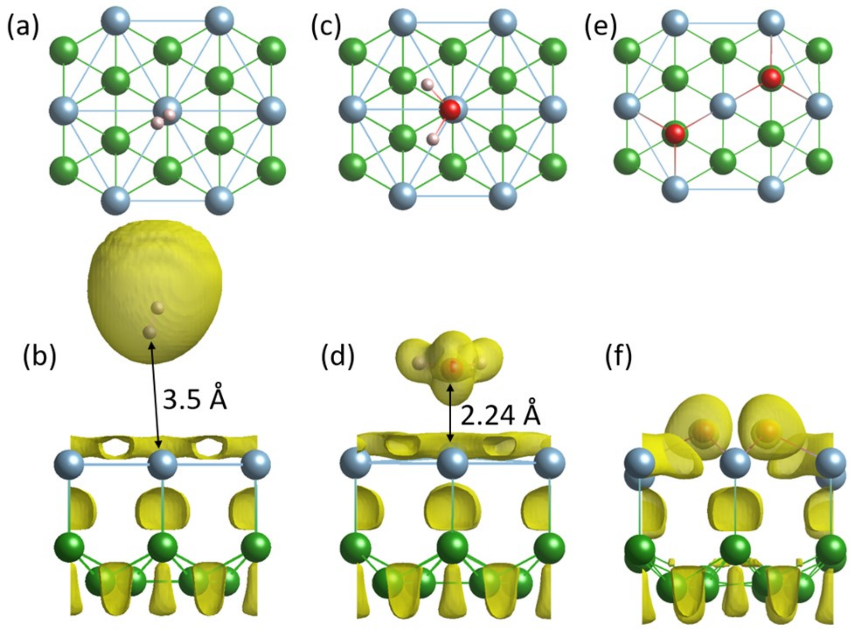

Fig. S4 (a, b, c) Molecule (H_2, O_2, H_2O) adsorption on the AlB_3 layer and (b,d,f) their corresponding ELF with the isovalue 0.75.

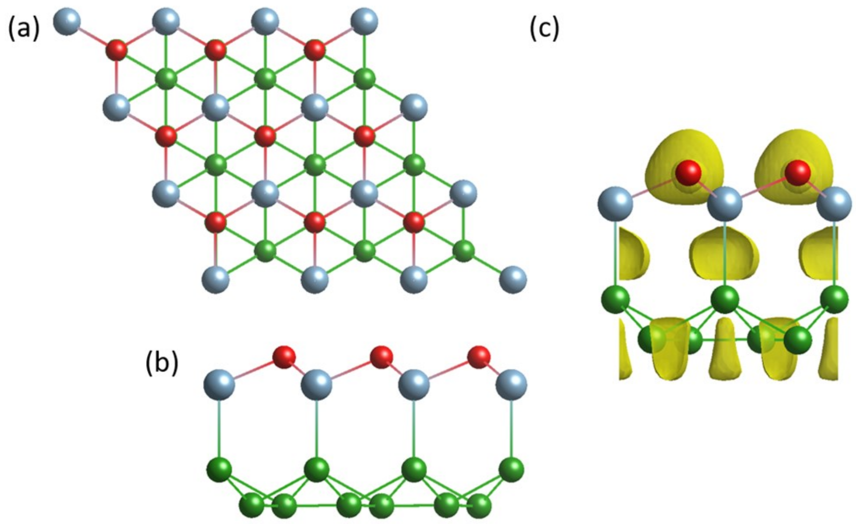

Fig. S5 (a-b) Geometry of fully oxidized AlB_3 heterosheet and (c) its ELF with the isovalue 0.75.

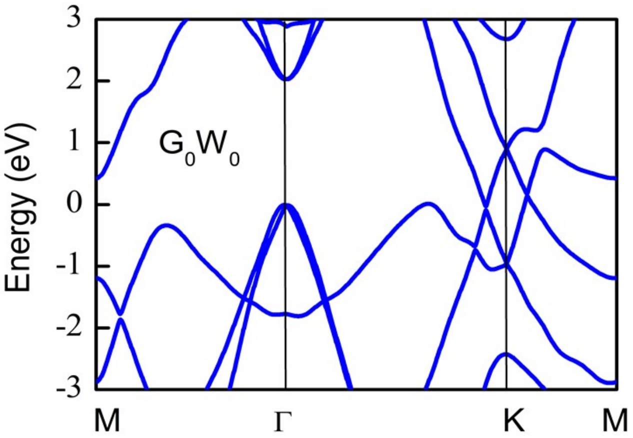

Fig. S6 Band structures of the AlB_3 heterosheet by G_0W_0 method.

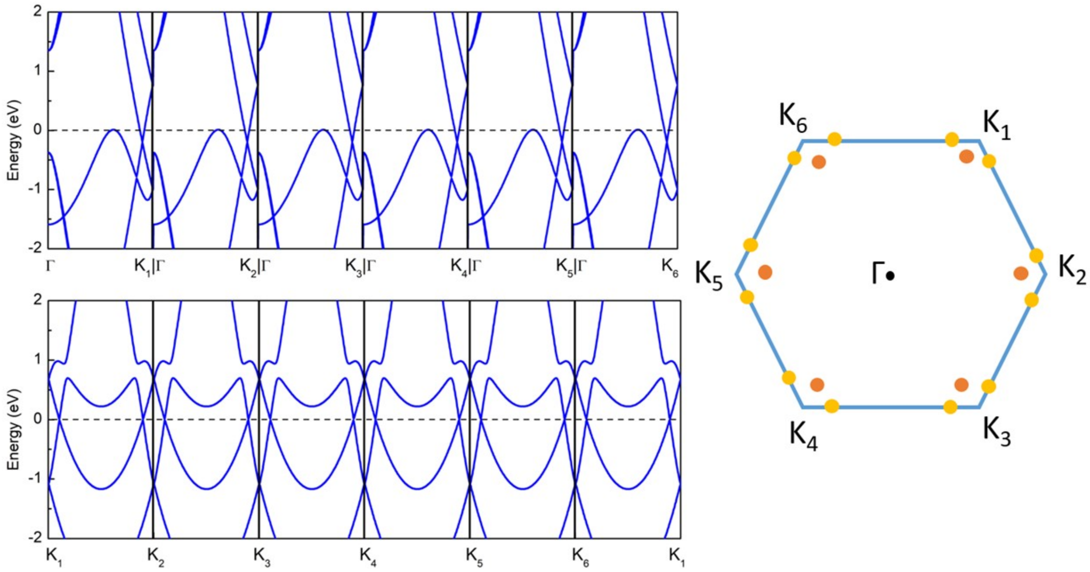

Fig. S7 Band structures of the AlB_3 heterosheet along selected K point paths.

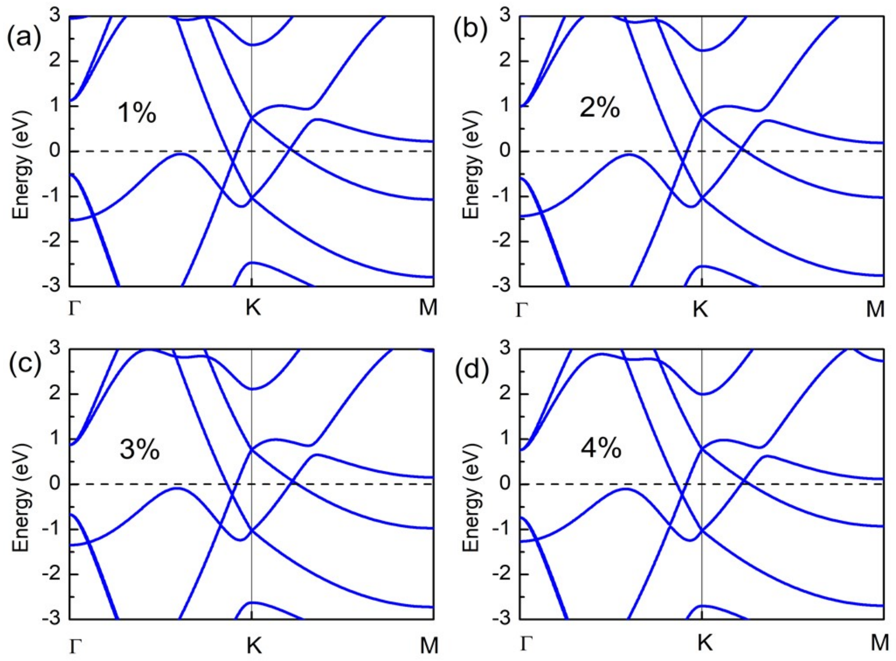

Fig. S8 Band structures of the AlB_3 heterosheet when biaxial strain was applied.

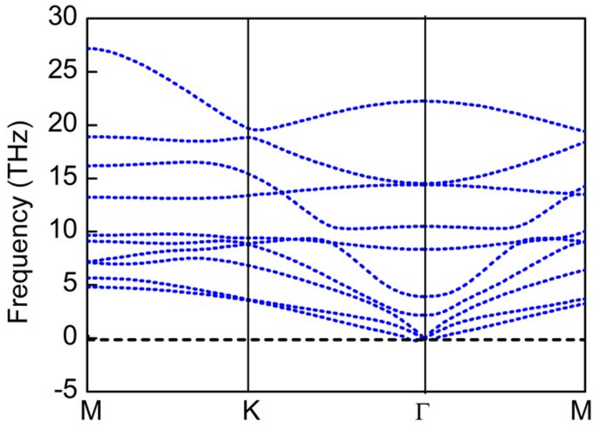

Fig. S9 The phonon band dispersion of AlB_3 heterosheet.

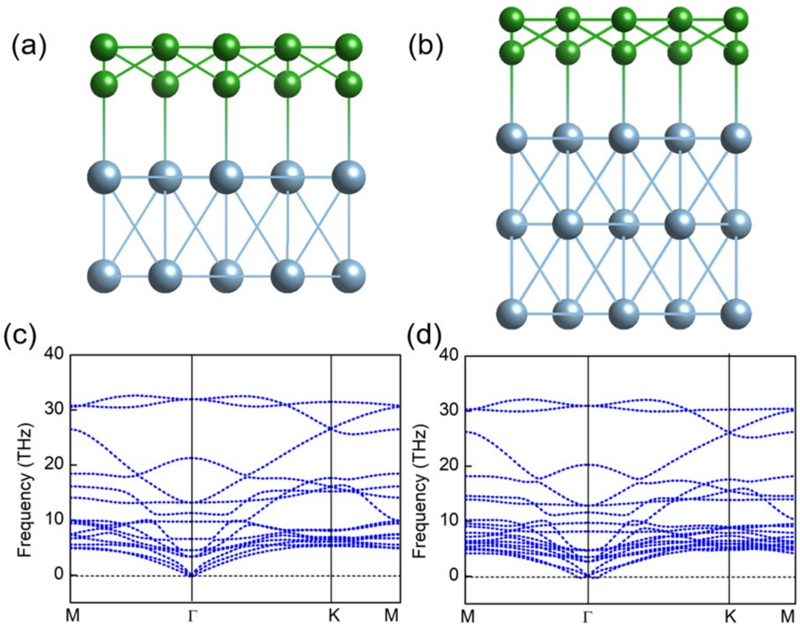

Fig. S10 (a-b) Side view of the bilayer/trilayer Al + borophene system and (c-d) their corresponding phonon spectrum.

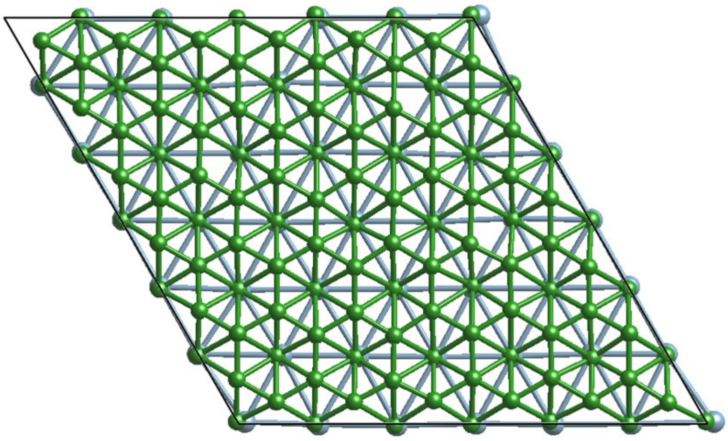

Fig. S11 The AIMD snapshot of AlB_3 heterosheet at 300 K after 10 ps.

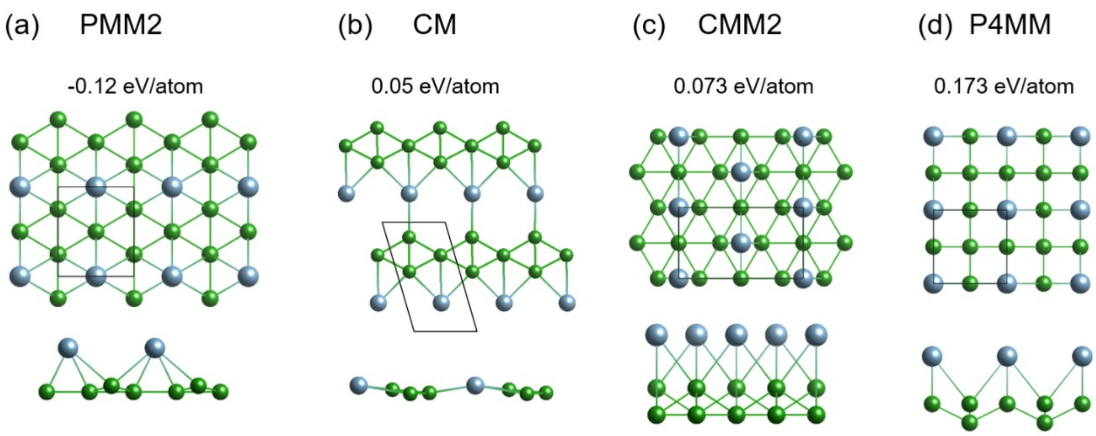

Fig. S12 (a-d) Top and side views of the AlB_3 allotropes obtained from structure search. The space group and relative energy with respect to the AlB_3 heterostructure in the main text (P6MM phase) are presented as well.

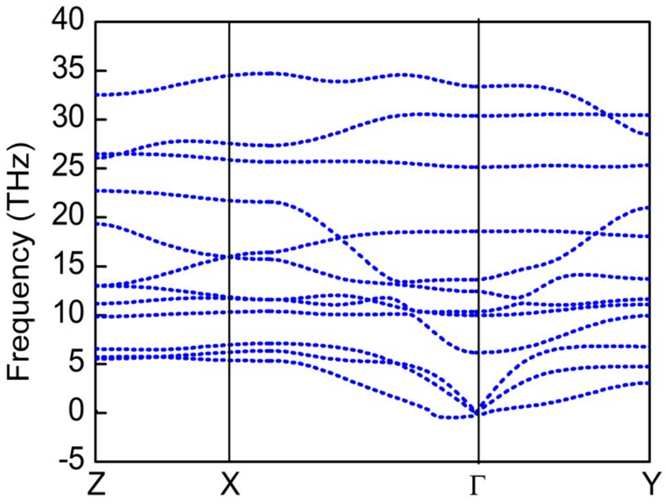

Fig. S13 Phonon spectrum for the PMM2 AlB_3 sheet, calculated using a (6x4x1) super cell. The plot shows the remainder of an imaginary branch that shrinks from smaller to larger super cells and that we attribute to the self-interaction of the displaced atom.

Table S1. The elastic constants (in GPa · nm) of the AlB_3 heterosheet.

**Phase**	C_{11}	C_{22}	C_{66}	C_{12}
AlB_3	269.45	270.88	115.6	40.42
